# Noradrenergic Control of Gene Expression and Long-Term Neuronal Adaptation Evoked by Learned Vocalizations in Songbirds

**DOI:** 10.1371/journal.pone.0036276

**Published:** 2012-05-04

**Authors:** Tarciso A. F. Velho, Kai Lu, Sidarta Ribeiro, Raphael Pinaud, David Vicario, Claudio V. Mello

**Affiliations:** 1 Department of Behavioral Neuroscience and Neurological Sciences Institute, Oregon Health and Science University, Portland, Oregon, United States of America; 2 Psychology Department, Rutgers University, Piscataway, New Jersey, United States of America; 3 Brain Institute, Federal University of Rio Grande do Norte, Natal, Rio Grande do Norte, Brazil; INSERM/CNRS, France

## Abstract

Norepinephrine (NE) is thought to play important roles in the consolidation and retrieval of long-term memories, but its role in the processing and memorization of complex acoustic signals used for vocal communication has yet to be determined. We have used a combination of gene expression analysis, electrophysiological recordings and pharmacological manipulations in zebra finches to examine the role of noradrenergic transmission in the brain’s response to birdsong, a learned vocal behavior that shares important features with human speech. We show that noradrenergic transmission is required for both the expression of activity-dependent genes and the long-term maintenance of stimulus-specific electrophysiological adaptation that are induced in central auditory neurons by stimulation with birdsong. Specifically, we show that the caudomedial nidopallium (NCM), an area directly involved in the auditory processing and memorization of birdsong, receives strong noradrenergic innervation. Song-responsive neurons in this area express α-adrenergic receptors and are in close proximity to noradrenergic terminals. We further show that local α-adrenergic antagonism interferes with song-induced gene expression, without affecting spontaneous or evoked electrophysiological activity, thus dissociating the molecular and electrophysiological responses to song. Moreover, α-adrenergic antagonism disrupts the maintenance but not the acquisition of the adapted physiological state. We suggest that the noradrenergic system regulates long-term changes in song-responsive neurons by modulating the gene expression response that is associated with the electrophysiological activation triggered by song. We also suggest that this mechanism may be an important contributor to long-term auditory memories of learned vocalizations.

## Introduction

The noradrenergic system modulates attention and alertness [1], [2] and is required for long-term memory consolidation and recall [3], [4], [5], [6], [7]. Mounting evidence indicates that the noradrenergic system acts by modulating both gene expression and electrophysiological activity. The locus coeruleus (LoC), the brain’s main source of noradrenergic input, is required for the cortical expression of plasticity-related immediate early genes induced by sensory stimulation [8], [9]. In addition, the LoC has been implicated in long-term electrophysiological changes following neuronal activation. For example, LoC stimulation induces long-term potentiation (LTP) of the hippocampal dentate gyrus, an effect that is markedly reduced by norepinephrine depletion [10], [11]. Thus, the role of the noradrenergic system in long-term memory appears to involve the modulation of both activity-induced gene expression and electrophysiological responsiveness of neuronal cells.

The auditory stimulation of zebra finches with species-specific song can induce robust and lasting brain changes in gene expression and in electrophysiological responsiveness to song stimuli. Birdsong thus provides a unique opportunity to investigate the role of noradrenergic transmission in modulating gene expression and electrophysiological plasticity. Zebra finches are representative songbirds, an avian order where vocalizations are learned based on auditory feedback [12]. When finches hear song, activity-dependent immediate early genes like *zenk* (aka *egr-1*, *zif-268*) and *arc*, which are involved in memory consolidation and synaptic plasticity [13], [14], are rapidly induced [15], [16]. This effect is most pronounced in the caudomedial nidopallium (NCM), a high order auditory area analogous to supragranular layers of the mammalian auditory cortex [17] and involved in the perceptual processing and memorization of song [18]. Song-induced gene expression is higher for conspecific songs than for other sounds [15], [16], and it is modulated by novelty, behavioral state, context and experience [19]. NCM also shows robust song-evoked electrophysiological responses that decrease upon repeated stimulus presentations [20]. This decrease is a form of long-lasting song-specific adaptation, since responses are restored by a novel song. Local blockade of RNA or protein synthesis immediately after song stimulation disrupts long-term adaptation without interfering with its acquisition. Thus, song-induced genes seem to be required for long-term adaptation, possibly representing a critical link between neuronal activation and long-term changes underlying song perceptual learning [21], [22]. Nonetheless, mechanisms controlling song-induced gene expression and adaptation remain undefined.

We reasoned that the noradrenergic system could directly regulate song-induced gene expression, since the latter depends on novelty and attention. Here we show that NCM’s *zenk*-expressing neurons are close to noradrenergic terminals and express α-adrenergic receptors. We also show that local α-adrenergic antagonism interferes with song-induced gene expression without affecting spontaneous or evoked electrophysiological activity, thus dissociating the molecular and electrophysiological responses to song. Moreover, α-adrenergic antagonism disrupts the maintenance but not the acquisition of the adapted state. These findings implicate noradrenergic transmission in both electrophysiological and gene expression responses to birdsong, a learned auditory stimulus. We suggest that the noradrenergic system acts locally to modulate the transcriptional response induced by electrophysiological activity in a central auditory area, resulting in long-term adaptation of song-responsive neurons. This effect may reflect the involvement of the noradrenergic system in the formation of long-term perceptual memories for song and possibly other sensory stimuli at the cortical level.

## Methods

### Animals

All experiments used adult female zebra finches (*Taeniopygia guttata*) that were obtained from our own breeding colonies at the OHSU and at Rutgers University, or from local breeders. The birds were kept under a 12∶12 light:dark cycle; food and water were provided *ad libitum*. All procedures involving birds were approved by the Animal Care and Use Committees (IACUC) at the OHSU and Rutgers University, and are in accordance with NIH guidelines.

### Song Stimulation for Gene Expression Analysis

Birds were isolated for about 18 hr in sound-attenuated chambers (≈76×31×28 cm). They were then stimulated with playbacks of a medley of three different conspecific songs presented in succession (each for 15 sec followed by 45 sec of silence), at 70 dB mean SPL at 35 cm from the speaker (essentially as in [15]), for variable total durations according to the experiment. Controls consisted of birds that underwent the acoustic isolation and were not stimulated.

### Tissue Preparation for Gene Expression Analysis

For *in situ* hybridization, the birds were sacrificed by decapitation 30 min after the start of the stimulation, and their brains were quickly dissected from the skull, frozen in Tissue-tek (Sakura Finetek) in a dry ice/isopropanol bath, and stored at −80°C. Parasagittal 10 µm sections were cut on a cryostat, thaw-mounted onto slides, and stored at −80°C until use. For immunohistochemistry, birds were sacrificed by Nembutal overdose 90 min after the start of the stimulation, and perfused transcardially with phosphate-buffered saline (PBS), pH 7.4, followed by 4% paraformaldehyde in 0.1 M phosphate buffer (PB), pH 7.4. Brains were then dissected out, washed overnight at 4°C in 0.1 M PB, cryoprotected by placement in 20% sucrose in 0.1 M PB at 4°C, and frozen in embedding medium (TissueTek; Sakura Finetek) in a dry-ice/isopropanol bath. Parasagittal 20 µm sections were cut on a cryostat, and thaw mounted onto Superforst Plus slides (Fisher Scientific). Sections were dried out and stored at −80°C.

### Immunohistochemistry (IHC)

Brain sections were removed from the −80°C freezer and dried overnight at room temperature. Slides were then equilibrated for 30 min at room temperature in 0.1 M PB, followed by incubation for 30 min in blocking solution (0.5% skim milk and 0.3% TritonX-100 in 0.1 M PB). The slides were then incubated for 15 min in avidin solution and for 15 minutes in biotin solution at dilutions recommended by the manufacturer (Vector blocking kit; Vector Laboratories). Next, sections were incubated for 2 hours with one of the primary antibodies, either a rabbit anti-bovine-DBH (dopamine-beta-hydroxylase) antibody (1∶1,000; Eugene Tech), or a rabbit anti-egr-1 polyclonal antibody (Santa Cruz Biotechnology) that label respectively the DBH and ZENK proteins in the zebra finch brain [23], [24], followed by a 2 hour incubation with a biotinylated goat anti-rabbit immunoglobulin G (Vector Laboratories). Slides were then incubated for 2 hours in avidin-biotin complex (ABC) reagent (Vector Laboratories). Each of the steps above was followed by three washes (10 minutes each) in 0.1 M PB, pH 7.4. The slides were then developed by incubation in 0.03% diaminobenzidine (DAB), 0.15% nickel-ammonium sulfate, and 0.001%H2O2 in PB followed by rinsing in 0.1 M PB, dehydration, and coverslipping with Permount. To assess the specificity of our detection system (secondary antibody and ABC system), ICC controls were run as described above but omitting incubations with either the primary or secondary antibodies. For the double-labeling procedure (DBH plus ZENK detection), the sections were reacted sequentially for the DBH or ZENK IHC, but the ABC reagent used in the first reaction was inactivated by incubation in 0.3% H_2_O_2_ for 30 min to prevent cross reaction during the detection of the second antigen in the sequence**.** Importantly, since the two antigens have very different cellular localizations, the signals obtained with these two antibodies can be easily distinguished based on the cellular localization of the two proteins: ZENK is nuclear and DBH in this case is in neuronal processes.

### Clone Isolation for *in situ* Hybridization

The *zenk* and *hat2* (the songbird homolog of n-chimaerin) probes were derived from the cloned canary homologues described previously [15], [25]. To isolate clones representing the zebra finch homologues of α−adrenergic receptors (ADRAs), we designed PCR primers for each ADRA subtype based on conserved domains within mRNA sequences from several species in GenBank and from sequences derived from the zebra finch genomic archives. The primers were used to amplify fragments from *ADRA1a* (forward: 5′ggggctgattggttttggggtttt3′; and reverse: 5′ttgagcaggcgcacggagaagt3′), *ADRA1b* (forward: 5′tcttttcagccgtggatgtg3′; and reverse: 5′tctgggcagccgtggatgcg3′), *ADRA1d* (forward: 5′gtgggcgtgggggtctttcctg3′; and reverse: 5′ggcggcggcggctcctt3′); *ADRA2a* (forward: 5′gaccgctactggtccatcac3′; and reverse: 5′agcgtgtaggtgaaraagaa3′) *ADRA2b* (forward: 5′cagccagcgcccttcccaacac3′; and reverse: 5′tgcaccacatcccaccaaagta3′), and *ADRA2c* (forward: 5′cagaacctcttcctggtgtc3′; and reverse: 5′tasaggctgtagctgaagaa3′). Standard PCR reactions were performed with DNA from a zebra finch cDNA library [26], [27]. Two transcripts (ADRA1a and 1b) were not represented in our cDNA library and we instead used genomic DNA for the PCR amplification with primers targeting only the coding region of exon 1. The amplified products were excised from an agarose gel, eluted using Qiagen Gel Extraction Kit® (Qiagen Inc.), inserted into pPCRScript (Stratagene Inc.) and used to transform bacterial cells following standard procedures. Insert identity was confirmed by sequencing and analysis using DNAStar software (DNAStar Inc) and BLAST searches (See supplementary Table S1 for details).

### Probe Labeling for *in situ* Hybridization

Plasmid DNA for all clones analyzed was isolated using Qiagen Miniprep Kit (Qiagen Inc.), linearized with the appropriate restriction enzymes, and purified with Qiagen PCR Purification Kit (Qiagen Inc.). For radioactive procedures, we synthesized sense and antisense ^33^P-labeled riboprobes as described in [28]. For non-radioactive procedures, we prepared digoxigenin-(DIG) labeled ribroprobes as described in [16]. All riboprobes were purified in Sephadex G-50 columns and analyzed using a liquid scintillation counter or by visual inspection of a denaturing formaldehyde-agarose gel. Hybridization of sense riboprobes served as specificity controls for the *in situs* and yielded no detectable signal under our conditions (not shown).

### Radioactive *in situ* Hybridization (ISH)

ISH using ^33^P-labelled riboprobes for *zenk* and *hat2* was performed as previously described [28]. Hybridized slides were exposed to a phosphorimager screen or processed for emulsion autoradiography followed by Nissl counterstaining. The hybridizations and washes for all probes analyzed were performed at 65°C.

### Double *in situ* Hybridization (dISH)

We combined the procedures for radioactive (for each *ADRA*) and fluorescent (for *zenk*) ISH, followed by nuclear counterstaining (Hoechst) and emulsion autoradiography to reveal the radiolabeled probe. The overall protocol was essentially as described in [29] and [30].

### Systemic Injections of Noradrenergic Antagonists

Birds were isolated overnight in sound-attenuated chambers. In the following morning, they received an intramuscular (IM) injection of either a noradrenergic antagonist (phentolamine or propanolol) or vehicle (saline) and were immediately returned to the isolation chamber. Song playbacks started 10 minutes after this initial injection and lasted for 30 minutes. To ensure the effectiveness of the drug throughout the stimulation period, birds also received a second IM injection 10 minutes after stimulus-onset. At the end of the stimulation, animals were quickly sacrificed and their brains processed for *in situ* hybridization.

### Head Post Implants

For stereotaxic manipulations in the awake restrained preparation, birds were surgically implanted with head posts. For this purpose, birds were anesthetized with 50–55 mg/Kg IM of sodium pentobarbital, and placed in a stereotaxic apparatus containing a head post adapter (MyNeuroLab, Stereotaxic Head Holder for Birds). A round window was made on the skull by removal of the superficial layer of bone over the area that includes the bifurcation of the midsagittal sinus and NCM. A chamber was formed and a head post was attached to the skull using dental cement (Dentsply International Inc.). To allow for full recovery from anesthesia, further procedures were performed 24–48 hr after surgery.

### Acclimation Sessions

In order to minimize procedural induction of activity-dependent genes in the microinjection experiments for gene expression analysis, the birds were subjected to several acclimation sessions starting 24 hr after the head post implantation. Each session consisted of gently immobilizing the awake bird in a comfortable plastic tube, fixing the head post to the stereotaxic apparatus, maintaining the bird in the apparatus for 10 min (in the presence of a researcher), and returning it to the acoustic isolation chamber. Four such sessions (once every 2 hr) were performed on the day preceding the brain microinjections, and one last session on the day of the injections. The birds were kept in acoustic isolation chambers at all times, except during the acclimation sessions and microinjections. Both the acclimation and microinjections were performed in a sound-attenuated room with minimal sound exposure.

### Brain Microinjections

Four hours after the last acclimation session the birds were immobilized and their heads attached to the stereotaxic apparatus as in the acclimation session. A glass pipette was lowered into NCM through an opening in the bone (AP: 0.5 mm; ML: 0.65 mm; DV: 1.2 and 1.6 mm). Phentolamine mesylate (30 mM in saline**;** Sigma Aldrich) was injected (3 µmoles total; 100 nl/hemisphere over 2 min) into one hemisphere and vehicle in the contralateral hemisphere, using a hydraulic microdrive (Narishige International Inc.). To facilitate the identification of the injection site, the pipettes were coated with fluorescent latex beads (Lumafluor Corp.). The birds were then released from the apparatus and returned to the isolation boxes. Starting 10 min after the injections, the birds were stimulated with conspecific song for 10 min and sacrificed by decapitation 30 min after stimulation onset. The brains were then processed for ISH for *zenk* and *hat2*. In this experiment, we used the lowest dose of antagonist that gave a significant response in pilot experiments. Specifically, we tested 1.5, 3 and 15 µmoles of phentolamine mesylate and chose the 3 µmoles dose based on the ability to block gene expression. Although it is possible that higher doses may have an effect on the electrophysiological responses, we used in the electrophysiology experiments the lowest dose that effectively blocked gene expression.

### Densitometry

Phosphorimager autoradiograms of sections hybridized for *zenk* and *hat2* were obtained with a Typhoon 8600 (Molecular Dynamics Inc.) and quantified using NIH’s ImageJ software. Densitometric measurements taken over specific brain regions were subtracted for slide background and resulting values averaged for two adjacent sections. For normalization, all densitometric values were divided by the average values from unstimulated controls. The values obtained in the injected areas of vehicle- and phentolamine-injected hemispheres of song-stimulated birds were normalized by the respective values from non-stimulated control birds. To examine the effect of drug treatment, we used ANOVA followed by Fisher’s PSDL *posthoc* tests for pairwise comparisons, and a probability level of <0.05 for significance.

### Cell Counts

For quantitative analysis of double ISH, we used Neurolucida software (MicroBrightField Inc.) and estimated the number of labeled cells in specific areas of brain sections through NCM of song-stimulated birds (n = 3). We first placed a counting square of 200 X 200 µm over dorsal or ventral NCM, equidistant from NCM’s rostral and the caudal boundaries (L2a and the ventricle, respectively). We then centered a counting circle (12 µm diameter) over each fluorescent-labeled *zenk*-expressing cell. We next counted the number of emulsion autoradiography grains per labeled cell. We note that the typical nuclear diameter of most cells counted was in the 6–10 µm range and that the diameter of the counting circle was determined to be inclusive of the majority of grains associated with the cells. Cells with grain counts of at least 2 standard deviations above background levels (measured over the glass or over the neuropil between labeled somata) were considered *ADRA*-positive.

### Electrophysiological Recordings

One day after the head post implantation surgery, each bird was acoustically isolated for a day. Electrophysiological recordings then began 48 hr after the initial surgery, allowing for full recovery from anesthesia. Recordings were made in an acoustically isolated sound booth. The awake animal was immobilized in a comfortable tube and the head post was fixed to a stereotaxic device. A square grid with holes spaced at 250 µm intervals (Electron Microscopy Sciences, Fort Washington, PA) was cemented to the inner layer of the skull over a craniotomy that exposed the brain surface in order to define the position of electrode penetrations along the caudo-rostral and medio-lateral axes. Recordings were made at 7 sites bilaterally (3 in the left hemisphere, 4 in the right) using seven quartz-platinum/tungsten microelectrodes (1–4 MOhms impedance), controlled by a multielectrode microdrive (Eckhorn model, Thomas Recording). White noise stimuli with the amplitude envelope of canary song were presented to search for responsive sites that indicated the dorsal border of NCM.

### Testing the Short-term Effect of Phentolamine Microinjection on Electrophysiological Responses in NCM

After responsive sites were found, the bird was trained by presenting a set of 3 novel conspecific songs (recorded from animals that the subject bird had never heard before), each repeated 100 times in a block at an inter-stimulus interval of 8 sec. Multi-unit recordings (filters: high pass, 500 Hz, low pass 5 kHz) were made through the microelectrodes while song stimuli were played through a speaker (0.5 m from the bird’s ears) at an amplitude of 70 dB SPL, under computer control (Spike2 version 5.18, Cambridge Electronic Design).

After the training was completed, a glass micropipette (tip I.D. ∼30 mm) was driven into the right hemisphere through an adjacent grid hole to approximately the same depth as the electrodes, and phentolamine (phentolamine mesylate, Sigma-Aldrich, 300 mM in distilled water) was administered with a microinjector system (Drummond Scientific Wiretrol) advanced by a hydraulic microdrive (Narishige). Thus, drug microinjections were made unilaterally while recordings were made bilaterally, with the contralateral hemisphere used as control for trends in activity and responsiveness over the course of the experiment. Each animal received an initial loading dose (30 nl injected over 1.5 min), followed by maintenance doses (2 nl every 1 min) for the duration of the auditory stimulus trials. Auditory responses were recorded during playback of a set of 25 repeats of each of 6 conspecific songs (the 3 previously trained songs (A2) and 3 novel songs (B1)), presented in shuffled order.

At the conclusion of recording, three small electrolytic lesions were made in each hemisphere to enable histological reconstruction of recording sites. Animals were then given a lethal dose of anesthetic and perfused with saline, followed by paraformaldehyde. Their brains were cut into 50 µm sections that were stained with cresyl violet. The lesion sites were confirmed histologically and then the other recording sites were located through their relative distance to the confirmed lesion sites, using grid position and depth coordinates. NCM is located within the caudo-medial area of the forebrain and has larger, more loosely packed cells compared to field L, located rostral to NCM (Fortune and Margoliash, 1992). Recording sites found to be in field L2 and the transition areas between field L2 and NCM were excluded from analyses.

### Testing the Long-term Effect of Phentolamine Microinjection on Electrophysiological Responses in NCM

After responsive sites were found, a glass micropipette filled with phentolamine was driven through a grid hole that targeted one of a cluster of responsive sites in one hemisphere to approximately the same depth as the electrodes. These experiments used a 150 mM concentration of phentolamine, lower than in short- term experiments, but the difference in duration of the injection period caused the total dose of drug to be approximately the same in both the short- and long- term experiments. Each animal received an initial loading dose (30 nl in 1.5 min), followed by maintenance doses (6 nl every 1 min) for 30 min. The same amount of vehicle was injected into the equivalent position in the contralateral hemisphere as control. Three birds received phentolamine in the right hemisphere and 3 received phentolamine in the left hemisphere. Each bird was then returned to its cage and, after a 10 min interval, trained with playback of a series of 8 conspecific training songs, each repeated 200 times at an interval of 8 sec.

The next day, 20–21 hr after song training, the bird was again positioned in the stereotaxic apparatus. Electrodes were lowered into each hemisphere at the same grid positions as in the initial recordings. Recordings were made while playing 16 songs (8 training songs heard just after the injections on the previous day and 8 novel songs). Twenty-five repeats of each song in a shuffled order were presented in 4 groups of 4 songs each (2 training and 2 novel songs). At the conclusion of recording, three small electrolytic lesions were made in each hemisphere and the recording sites were confirmed histologically in NCM, as described above.

### Electrophysiological Data Analysis

In order to enable valid comparisons between conditions (before vs. during drug administration), multi-unit responses were recorded at each site because these responses are relatively stable over a period of 1–2 hr compared to single-units, which cannot reliably be held for the necessary time period in the awake preparation. Recording sites that showed large changes in spontaneous activity assessed during the control period (see below) were not included in the analysis.

Multi-unit neural responses to song stimuli were quantified as the difference between the root-mean-square (RMS) values obtained for each electrode during a response window (from stimulus onset to stimulus offset plus 100 ms) and the RMS during the control period of each trial (a 500 ms window occurring prior to stimulus onset). To compute the RMS, each digitized value is squared, the mean of these squares over the response interval is computed, and the square root of that mean is taken. This provides a method of rectifying the multi-unit activity and computing its average power.

In short-term experiments, response amplitudes (averaged across sites in each hemisphere) to the last trials (trials 21–25) of each song from set A heard before injection were compared to responses to the first trials (trials 2–6) of the same set of songs heard during phentolamine injection. The very first trial was excluded from analysis because of its variability. If phentolamine affects established short-term adaptation, the response level would change on the injected side. For this analysis, the ratio of the response amplitude to the same songs before and after the drug injection were averaged across sites in each hemisphere and compared with the Wilcoxon matched pairs test.

A second analysis compared averaged response amplitudes in each hemisphere for a set of novel songs B during drug injection. Response amplitudes were measured as the averaged responses to the first trials (trials 2–6) of each stimulus. Then the amplitudes obtained from the two hemispheres were compared. Then, in order to test whether phentolamine could block the process of adaptation, the responses to the last trials (trials 21–25) of the same songs were measured and the ratios of these values to the amplitude measured to the first trials (trials 2–6) of the same songs were taken and compared between hemispheres.

Spontaneous activity was also measured at each site as the mean of the RMS amplitude over a baseline period (0.5s) preceding the stimulus on each trial. The ratios of spontaneous activity before and during phentolamine injection were computed and averaged across each hemisphere for comparison.

For each comparison, the Wilcoxon test was used when data from electrophysiology experiments was compared across birds (9 birds). We also used ANOVA to analyze the same data sets, using samples from individual sites, after verifying normality of distributions and homogeneity of variances with the Kolmogorov-Smirnov and Levene’s test, respectively. The ANOVAs used factors of Side (drug vs. control) and Condition (before vs. during drug injection) as a repeated measure, and their interaction. The criterion for statistical significance was set at p<0.05, two-tailed. Statistical computations were carried out in Statistica (Statsoft).

The strength of the long-term neuronal memory for any song can be measured by presenting that song in a shuffled set with novel songs and computing a familiarity Index (FI). The FI for a given test song is the ratio of the mean adaptation rate for the novel songs to the adaptation rate for the test song at the same site [31]. The adaptation rate for each song at each site was determined by an established method [31]. The slope of the regression between response amplitude and repetition number over the linear portion of the adaptation trajectory (trials 6–25) was normalized by dividing it by the mean response amplitude over the same responses to produce an adaptation rate. The rate represents the percentage drop in response amplitude per stimulus repetition. If the test song is not familiar, its adaptation rate will be similar to the rate for novel songs and the FI will be near 1.0. If the test song is familiar, its adaptation rate will be lower than rates for novel songs and the FI will be greater than 1.0 [20], [31]. In testing the long term effect of phentolamine injection in NCM during training, the FI for each training songs was computed at each site, then averaged across sites in each hemisphere. The Wilcoxon matched pairs test was used to compare adaptation rates and FI values between the hemispheres.

## Results

### Noradrenergic Innervation of NCM

**Figure 1:**
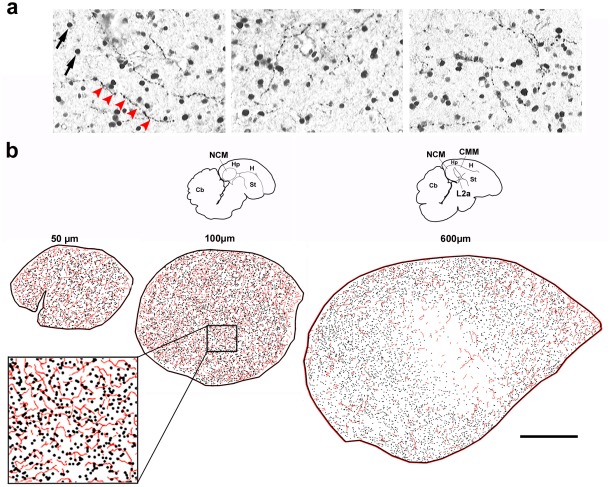
Noradrenergic innervation and sensory-activated neurons in NCM. a) Representative images of double-immunostaining for ZENK (nuclear) and DBH (fibers) in parasaggital brain sections of song-stimulated zebra finches; red arrowheads depict a DBH-positive fiber. b) Camera lucida drawings from serial brain sections depicting DBH-positive fibers (red traces) and ZENK-expressing neurons (black dots) at different medial to lateral NCM levels (in µm from the midline); NCM location is indicated by the small brain diagrams on top and the inset at the bottom left shows a detailed view of the tracings, at about half the magnification as the panels in a; the brain section containing the most medial NCM (50 µm from the midline) is often incomplete and therefore is not represented here. Abbreviations: Cb, cerebellum; CMM, caudomedial mesopallium; Hp, hippocampus; H, hyperpallium; L2a, subfield L2a of field L; NCM, caudomedial nidopallium; St, striatum. Scale bar, 500 µm.

We have previously used immunostaining for the noradrenergic marker dopamine β-hydroxilase (DBH) to show that NCM contains noradrenergic fibers [23]. To further investigate the noradrenergic innervation of NCM and how it relates to song-induced gene expression, we double immunostained serial parasagittal brain sections from song-stimulated birds using antibodies against the noradrenergic marker dopamine beta-hydroxylase (DBH) and ZENK protein. We observed that ZENK-expressing neurons as revealed by nuclear staining (Fig 1a, arrows) are in close proximity to DBH-stained fibers (Fig 1a, red arrowheads). Camera lucida reconstructions of the DBH and ZENK staining revealed a relatively dense network of both noradrenergic fibers (Fig 1b, red traces) and ZENK-expressing cells (Fig 1b, black dots) throughout NCM. The density of labeling for both markers decreased from medial to lateral levels (Fig 1b, left to right), particularly at a centro-rostral region corresponding to field L2a, which lacks song-induced ZENK expression.

### Noradrenergic Modulation of *zenk* Expression in NCM

**Figure 2:**
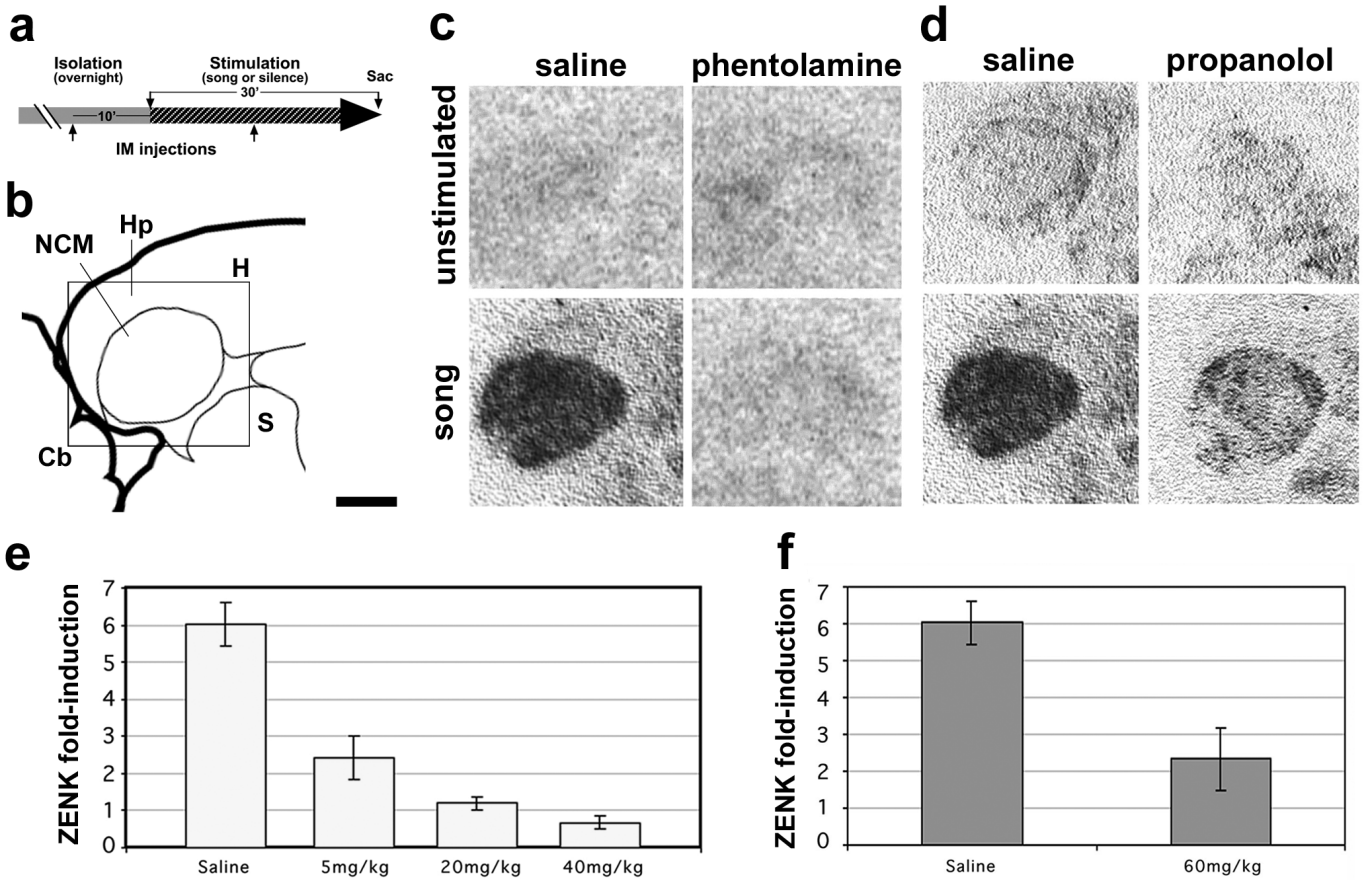
Systemic injections of α-adrenergic receptors antagonists block song-induced gene expression. a) Schematic representation of the experimental design depicting the timing of systemic injections (10 min prior to and 10 min after stimulation onset), and the overnight sound isolation (grey bar) and stimulation periods (30 minutes, patterned bar); b) Camera lucida drawing of a parasagittal brain section containing NCM at the level analyzed. c and d) Autoradiographic images of brain sections from unstimulated controls and song-stimulated birds systemically injected with saline or phentolamine (c) and saline or propanolol (d), hybridized with *zenk* riboprobes. e–f) Song fold-induction estimates of *zenk* mRNA based on quantitative autoradiography in birds injected with phentolamine (e) or propanolol (f) compared to vehicle (saline); fold-induction values were calculated by dividing values in song-stimulated birds by unstimulated controls for each drug treatment. Abbreviations: Cb, cerebellum; Hp, hippocampus; H, hyperpallium; NCM, caudomedial nidopallium; S, septum. Scale bar: 1 mm.

To examine whether the noradrenergic system modulates the song-induced expression of activity-dependent genes in NCM, we administered α- or β-adrenergic receptor antagonists (phentolamine or propranolol, respectively) systemically to adult female zebra finches. Birds were sound-isolated overnight, received a first drug or vehicle (saline) IM injection 10 min prior to stimulation onset and a second dose 10 min after the onset of stimulation (Fig 2a), and were sacrificed at the end of the stimulation period (30 min). As expected, *in situ* hybridization (ISH) revealed marked *zenk* induction in the NCM of vehicle-treated birds (Fig 2c, compare top vs. bottom left panels). In contrast, *zenk* mRNA levels in song-stimulated phentolamine-injected birds were comparable to those in unstimulated controls (Fig 2c, compare top and bottom right panels; representative examples of birds injected with 20 mg/kg–52.8 µM – of phentolamine are shown), indicating that the systemic blockade of α-adrenergic receptors inhibits song-induced gene expression in the auditory telencephalon. A quantitative analysis showed a significant decrease in *zenk* induction in phentolamine-injected birds compared to saline injections (Fig 2e; Fisher’s PSLD F = 99.257; ANOVA, *p*<0.0001, n = 3 per group). Importantly, this reduction is observed at a relatively low dose (5 mg/Kg–13.2 µM -, compared to higher doses −3.15–10 mM range - found to be effective in blocking noradrenergic transmission when directly infused into the mouse olfactory bulb, [32]), and no significant differences are seen between control and stimulated birds at higher doses (Fisher’s PSLD, *p* = 0.83 for 20 and 40 mg/kg combined, n = 4 respectively). For propranolol, we observed only a partial decrease of song-induced *zenk* expression (Fig 2d and f; Fisher’s PSLD F = 67.345; ANOVA, *p*<0.001) at a very high dose (60 mg/Kg – 3 µM, compared to effective doses in the 10–300 nM range in various learning paradigms in other organisms, including inhibitory avoidance in chicks [33]) indicating a lesser involvement of this receptor subtype in modulating song-induced gene expression. These high doses of propranolol led to undesirable behavioral side effects such as light sedation and trembling that were absent in phentolamine-injected birds, thus we did not pursue higher doses or attempt to build a dose-response curve. In a pilot attempt to build a dose-response curve, lower doses of propranolol had no apparent effects on song-induced *zenk* expression (not shown).

**Figure 3:**
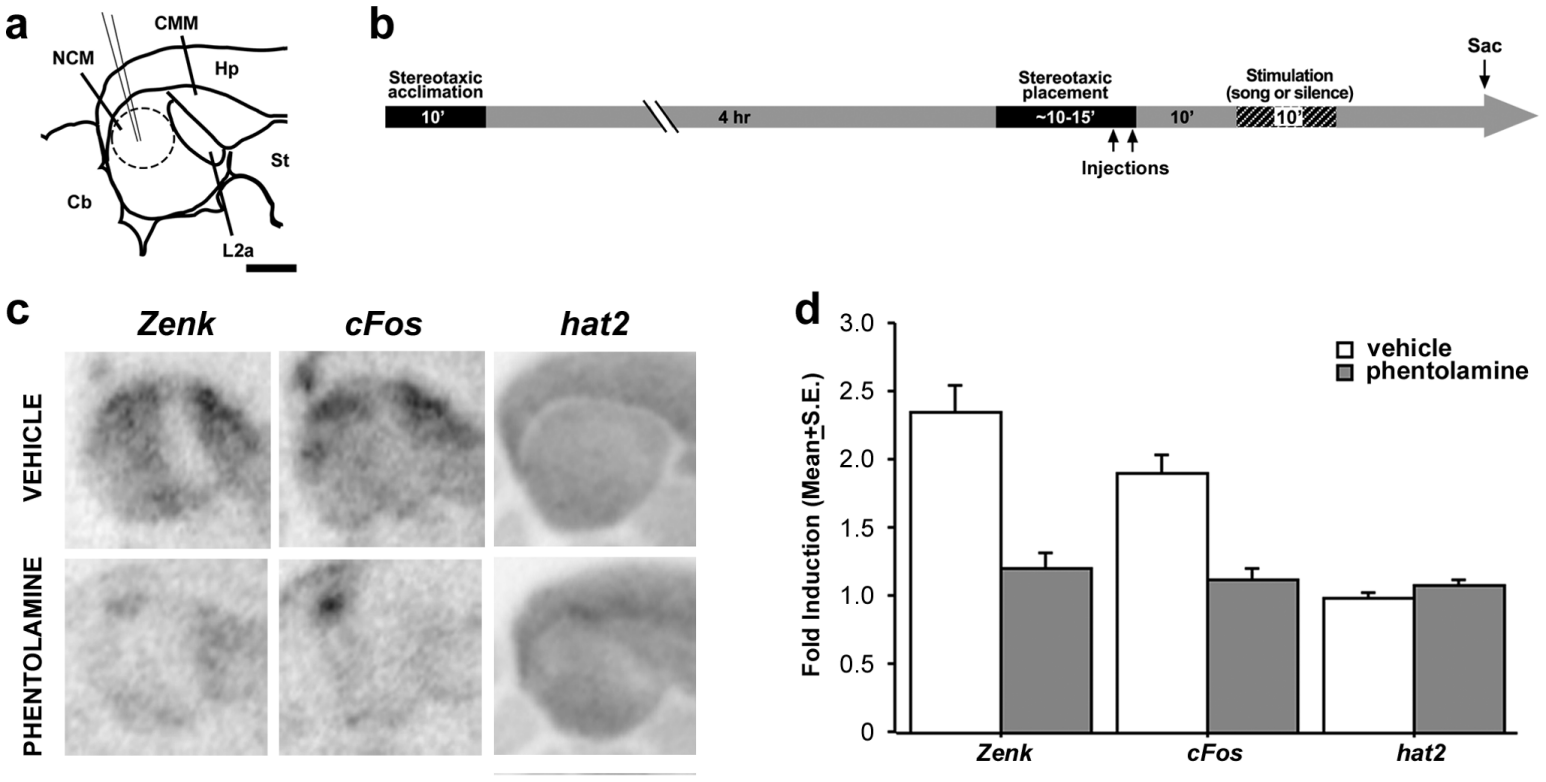
Local action of noradrenaline modulates song-induced gene expression in NCM. a) Camera lucida drawing of a parasagittal section containing NCM, field L2a and CMM (about 500 µm from the midline), indicating the site of injections (pipette and dashed circle). b) Schematic representation of the experimental design depicting the timing of stereotaxic injection into NCM of awake restrained birds in relation to silence and song stimulation periods. c) Autoradiograms of adjacent parasagittal sections from a representative bird that received an injection of phentolamine or vehicle (opposite hemispheres) into NCM, hybridized with *zenk*, *c-fos* or *hat2* riboprobes. d) Fold-induction values for *zenk*, *c-fos* and *hat2* in the NCM of vehicle- (white bars) or phentolamine-injected (gray bars) hemispheres in birds stimulated for 10 min with conspecific song normalized to the corresponding injected hemispheres in unstimulated controls. For abbreviations, see Figs 1 and 2. Scale bar in a: 2 mm.

To determine whether song-induced gene expression in NCM is dependent on local α-adrenergic transmission, we injected phentolamine into the NCM in one hemisphere of awake-restrained birds (Fig 3b), and vehicle (saline) in the contralateral hemisphere. Injected birds were then returned to their cages and presented with song playbacks (song-stimulated birds) or maintained in silence (unstimulated controls). ISH revealed marked hemispheric differences in the expression of *zenk* and *c-fos* in song-stimulated birds (Fig 3c, left and middle panels, respectively). In contrast, expression of *hat2* (the songbird homolog of n-chimaerin), a gene that is enriched in the songbird forebrain but not regulated by song [16], [25], [34], showed no apparent differences between phentolamine- and vehicle-injected hemispheres in the same birds (Fig 3c, right panels). Densitometric analysis revealed that *zenk* and *c-fos* were induced normally in saline-injected hemispheres of song-stimulated birds, reaching ∼2-fold induction when normalized to saline-injected hemispheres of unstimulated controls (Fig 3d, left and middle, white columns). In contrast, *zenk* and *c-fos* expression levels in phentolamine-injected hemispheres of song-stimulated birds did not show significant induction when normalized to the phentolamine-injected hemispheres of unstimulated controls (Fig 3d, grey columns; fold-induction ∼1), indicating that phentolamine injections abolished the song-induced expression of these mRNAs. In contrast, *hat2* expression analyzed in adjacent brain sections showed no induction in song-stimulated birds and no significant differences between vehicle- and phentolamine-injected hemispheres (Fisher’s PSLD F = 1.058; ANOVA, *p* = 0.41).

### Local α−adrenergic Blockade does not Affect Auditory Responses in NCM

**Figure 4:**
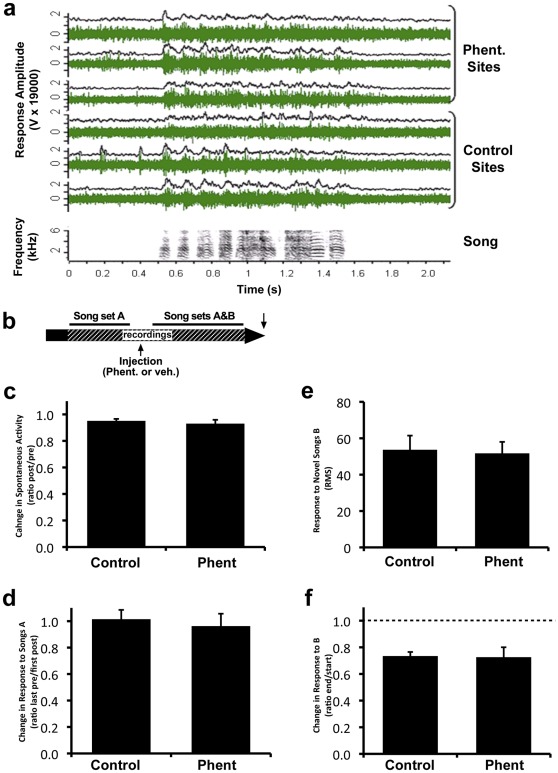
Alpha-adrenergic blockade does not affect short-term responses in NCM. a) Representative responses to a song stimulus shown as raw multi-unit activity (green), and as RMS (black, 20 ms bins) for 6 simultaneously recorded sites (3 in the phentolamine hemisphere, 3 in the control hemisphere). b) Schematic representation of the experimental design for short-term adaptation: initial playback of songs from set A followed by phentolamine injection and then playback of songs from both sets A and B in shuffled order; dashed bar indicates the period of recordings. c) The change in NCM spontaneous activity is plotted as the ratio of activity recorded before phentolamine injection to activity during injection for both the control and phentolamine-injected hemispheres. A ratio of 1 indicates no change. d) The change in response amplitude to songs in set A from the end of training to the beginning of testing after drug injection is plotted as a ratio for both control and phentolamine-injected hemispheres. A ratio of 1 indicates no change. e) Response amplitude to songs in set B when novel during drug injection is shown for both control and phentolamine-injected hemispheres. f) Change in response to songs in set B when novel and when familiar after training during phentolamine injection is plotted as a ratio for both control and phentolamine-injected hemispheres. Similar adaptation (ratio <1, dotted line) is seen in both hemispheres.

To test for possible immediate effects of α−adrenergic blockade on electrophysiological properties of NCM, we used methods previously developed to show drug effects on inhibitory neurotransmission in this brain area [35]. We inserted multiple microelectrodes simultaneously into NCM in both the left and right hemisphere of awake, restrained adult male zebra finches (n = 9), and then recorded multi-unit activity (Fig 4a) while microinjecting phentolamine continuously near the electrodes in one hemisphere, while the other side served as a control.

In order to assess the effect of α−adrenergic blockade on song-evoked responses, we played a set of novel songs (Song set A) to the bird before the injection and then played those same songs together with another set of novel songs (Song set B) during phentolamine injection (Fig 4b). We first tested for an effect of drug injections by computing the ratio of spontaneous activity before injection to the activity during injection (Fig 4c) and found no difference between hemispheres (Wilcoxon, n = 9, z = 0.296, p = 0.77). When we compared the mean level of spontaneous bursting activity (typical of NCM) before and during phentolamine injection, we saw only a small (5–7%) decrease in activity in both hemispheres. This change is significant, but did not differ between hemispheres and there was no interaction between factors (ANOVA Condition F = 15.6, p = 0.002; Side F = 1.47, p = 0.23; interaction F = 0.672, p = 0.42). Such decrease is probably due to small displacements of the electrode caused by introduction of the pipette into the brain and/or the passage of time.

We then calculated the ratio between the amplitude of multi-unit responses on the last 5 trials of set A and the response amplitude on the first trials of the same set A stimuli when these songs were repeated during phentolamine injection (Fig 4d). Again there was no difference in the ratios between hemispheres, indicating that the response level had neither increased (due to loss of adaptation; data not shown) nor further decreased, as a result of drug application (Wilcoxon, n = 9, z = 0.65, p = 0.51). Analysis of the site data also showed no significant effects (ANOVA before/after F = 0.65, p = 0.42; Side F = 0.05, p = 0.83; interaction F = 0.93, p = 0.34). Thus, the absence of a significant difference between hemispheres is not due to underestimating the sample size by averaging data for each bird.

We next measured the amplitude of auditory responses on the first playback trials of novel songs from set B during drug application (Fig. 4e) and found no difference between control and phentolamine-injected hemispheres (Wilcoxon, n = 9, z = 0.29, p = 0.76). Analysis of the site data also showed no significant effects (ANOVA one-way Side F = 0.29, p = 0.60). Finally, we examined the change in the response amplitude with stimulus repetition to set B by computing a ratio between the response on the last 5 trials and the trials 2–6 of the same songs (Fig 4f) and found no significant difference between the control and phentolamine-injected sides (Wilcoxon, n = 9, z = 0.18, p = 0.86). Responses showed adaptation, decreasing significantly (by ∼27%) in both hemispheres, (ANOVA Adaptation F = 48.5, p<0.0001; Side F = 1.68, p = 0.20; interaction F = 0.09, p = 0.76).

In summary, we found that the presence of phentolamine 1) did not cause any change in ongoing spontaneous activity in NCM; 2) did not affect adaptation established prior to drug injection; 3) did not affect responses to novel songs; and 4) did not prevent short-term adaptation from occurring normally. Thus, we did not detect any systematic changes attributable to drug injection. This suggests that the action of NE, in the range necessary for activity-dependent gene expression, at α−adrenergic receptors does not make a significant contribution to ongoing activity, auditory responses, or immediate adaptation.

### α−adrenergic Blockade in NCM Affects Long-term Adaptation in NCM

**Figure 5:**
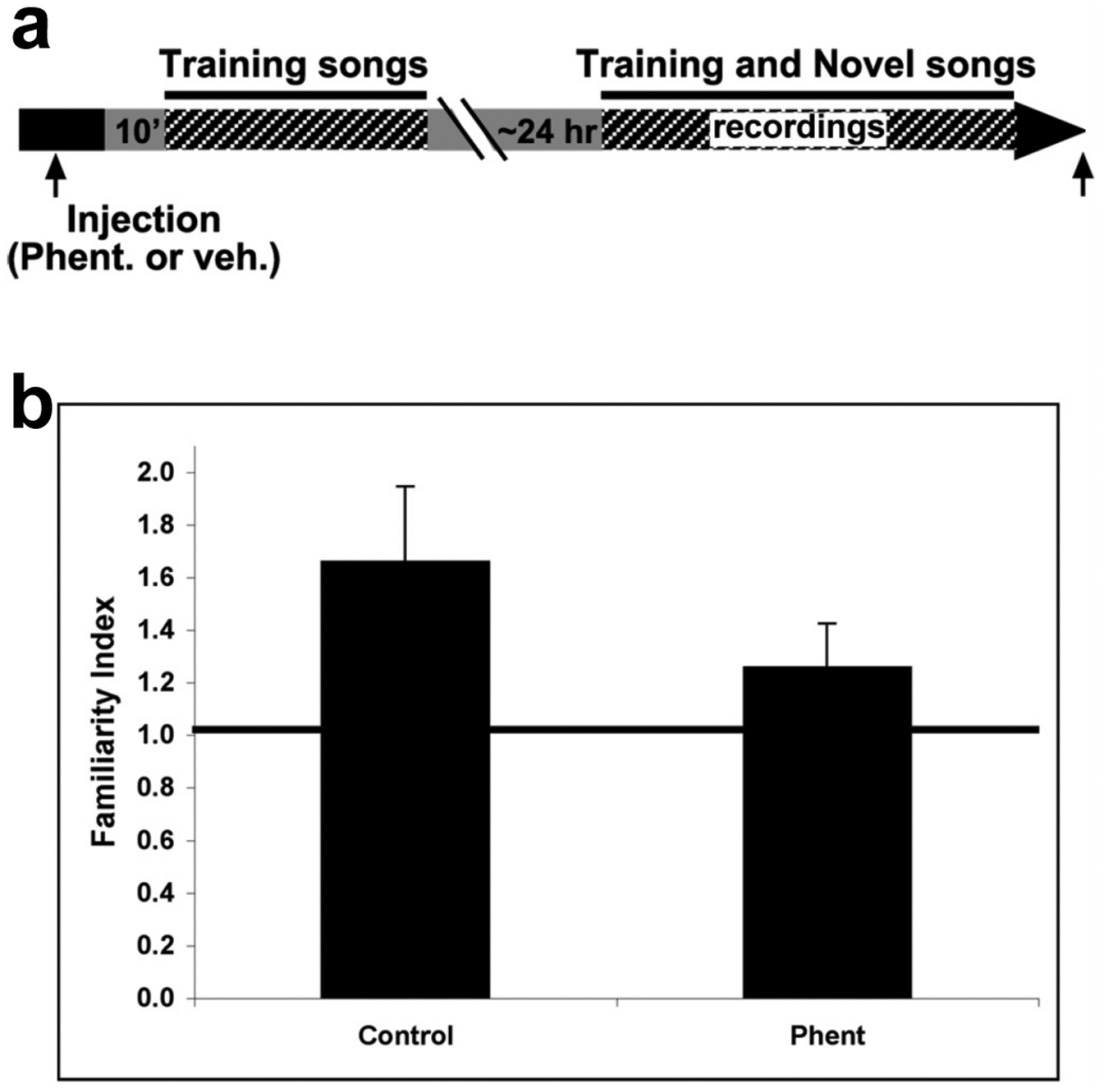
Alpha-adrenergic blockade disrupts long-term adaptation in NCM. a) Schematic representation of the experimental design depicting exposure to training songs followed the next day (20–21 hr later) by electrophysiological recordings during playback of both familiar training songs and novel songs in control and phentolamine-injected hemispheres. b) Familiarity indexes (the ratio between adaptation rates for novel songs and songs heard by the animal 20–21 h earlier, see Methods) are shown for both the control and phentolamine-injected hemispheres.

To assess the contribution of α−adrenergic modulation to long-term adaptation in NCM, we employed procedures that were previously used to demonstrate that new RNA and protein synthesis in NCM are required for long-term adaptation to be maintained for 40 hr or more [20]. We carried out preliminary recordings to locate NCM, then injected phentolamine into one hemisphere and vehicle into the other. We then presented a set of novel training songs. On the following day (20–21 hr later), we recorded from multiple microelectrodes placed in clusters around the injection site in each hemisphere while we played back the training songs together with a set of novel songs (Fig 5a).

Because stable recordings from the same multi-unit sites cannot be made over >20 hr to measure changes in response amplitude, we used the previously established method for assessing neuronal memory for songs, based on comparing the adaptation rates to test songs with rates for novel songs to compute a familiarity index (FI), as detailed in the Methods [20], [31]. An FI greater than 1.0 indicates that a test song is familiar in NCM. Using this method, we found that the FI for training songs in the control (vehicle-injected) hemisphere was significantly higher (Fig 5b) than in the experimental (phentolamine-injected) hemisphere (Control, 1.66 ± 0.28 vs. phentolamine, 1.26 ± 0.16, mean ± s.e.m.; n = 6, Wilcoxon Z = 2.20, *p* = 0.028). The adaptation rates to the novel songs used in computing the FI did not differ between hemispheres (Control -0.41 ± 0.08 vs. phentolamine −0.38 ± 0.07, mean ± s.e.m.; Wilcoxon, n = 6 birds, Z = 0. 31, *p* = 0.75) so the FI difference is due to the higher rates for training (familiar) songs in the phentolamine hemisphere. Training songs are clearly familiar for sites in the control hemisphere, but almost novel for sites in the phentolamine hemisphere. This result indicates that α−adrenergic blockade with phentolamine interferes with the maintenance of the initial adaptation to a repeated song over a long period. A necessary component of this process is located within the phentolamine diffusion zone that produces an effective blockade. We have not measured the extent of this zone, nor probed the range of concentrations necessary for effects in this area, but at least it does not extend to the opposite hemisphere, 1–1.5 mm away. Taken together, the findings in the short and long term experiments are consistent with a role for α−adrenergic modulation in long-term memory formation.

### Song-responsive NCM Cells Predominantly Express α-1-adrenergic Receptors

**Figure 6:**
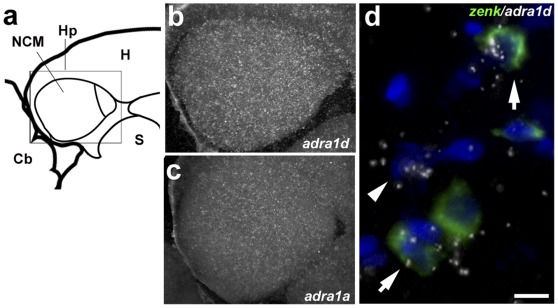
Expression of α-adrenergic receptors in NCM. a) Camera lucida drawing of a parasagittal brain section containing NCM and CMM (about 100 µm from the midline), indicating the position of the photomicrographs in b and c. b–c) Dark-field view of emulsion autoradiograms of brain sections hybridized with radioactively-labeled *ADRA1d* and *1a* riboprobes, respectively; shown is an area corresponding to the rectangle in a. d) High-magnification view of double *in situ* hybridization for *ADRA1d* (emulsion grains) and *zenk* (green fluorescence); arrows point to double-labeled cells and arrowhead indicates a single-labeled *ADRA1d*-positive cell.

The experiments above clearly established that α-adrenergic transmission plays important roles in the physiology of NCM. To start to address the cellular mechanisms associated with these actions, we next determined what α-adrenergic receptors (ADRA) are expressed in NCM. For this purpose, all known receptor subtypes (ADRA1a, 1b, 1d, 2a, 2b and 2c) were examined by *in situ* hybridization. Based on emulsion autoradiography, we found that *ADRA1d* is more strongly expressed in NCM compared to the other α-adrenergic subtypes (Fig 6b and c, *ADRA1d* and *1a*, respectively). Cellular analysis using double *in situ* hybridization revealed that although some *ADRA1d*-expressing cells are *zenk*-negative (Fig 6d arrowhead), *ADRA1d* is mostly expressed in *zenk*-expressing (song-responsive) neurons (6d arrows). Quantitative analysis (978 ZENK expressing cells from 3 different birds) showed that on average 64.8±3.9% of the *zenk*-expressing cells in NCM also express the *ADRA1d* subtype. This level of cellular co-expression is much (at least ∼3-fold) higher than those of all the other receptor subtypes (*1a*: 22.7±5.8%; *1b*: 14.6±5%; *2a*: 10.7±5%; *2b*: 18.8±3.1%; *2c*: 8.1±5%; n = 3 birds per gene). Altogether, these data give strong indication that the local noradrenergic transmission in NCM occurs predominantly through ADRA1d.

## Discussion

We have shown that local α-adrenergic transmission is required for song-induced gene expression, measured by the *zenk* reponse, and for the maintenance of long-term electrophysiological adaptation in central auditory neurons of songbirds. Our data also provide evidence that the gene expression and electrophysiological responses to song can be dissociated through local noradrenergic blockade, suggesting that the normal coupling of these responses is dependent on noradrenergic transmission. Our findings point to a noradrenergic involvement in cellular processes that have been postulated to subserve the memorization of birdsong, a learned vocal behavior. We suggest that this modulatory action of the noradrenergic system on the brain’s response to song may represent a general mechanism involved in the memorization of sensory events in the vertebrate brain.

### Noradrenergic Requirement for Activity-dependent Gene Expression

The locus coeruleus (LoC) is the brain’s main source of NE innervation [36]. LoC ablation using local administration of the neurotoxin 6-OHDA, or noradrenergic ablation using systemic administration of the neurotoxin DSP-4, dramatically reduced the cortical expression of the activity-dependent genes *zenk*/*zif-268* and *c-fos* in awake rodents [9]. Similarly, noradrenergic ablation by systemic administration of DSP-4 alters singing-induced *zenk* expression in the striatal song nucleus of zebra finches [37], and song-induced *zenk* expression in auditory areas of female canaries [38]. These observations suggest a regulatory role for NE in activity-dependent brain gene induction. Noradrenergic ablation with DSP-4 also appears to alter auditory responses in anesthetized zebra finches as measured by FMRI [39], and brain infusions with prazosin, an alpha-adrenergic inhibitor, alter the behavioral responses of female starlings to male songs [40]. However, because of widespread connections and actions of the LoC [41], it is unclear whether these effects of LoC lesions are related to a direct dependence on noradrenergic transmission or reflect changes in overall arousal/attention that could indirectly influence brain activity. Furthermore, neuromodulators that interact with NE (e.g., dopamine) may be affected by noradrenergic depletion and thus contribute to the effects of LoC ablation.

Our local application of phentolamine yielded direct evidence that intact α-adrenergic transmission in an auditory telencephalic area (NCM) is required for song-induced expression of activity-dependent genes. Because of the dense noradrenergic innervation of NCM, this effect was likely due to the blockade of α-adrenergic receptor (ADRA) activation by NE released from noradrenergic terminals. Indeed, we have observed that when NE is directly applied to NCM it can induce *zenk* locally in a dose-dependent manner (Supplementary Fig. S1), indicating that adrenergic receptors are present in NCM neurons and that their activation by NE can lead to the induced expression of *zenk*. Furthermore, although we cannot completely rule out a contribution of β-adrenergic transmission, the relative ineffectiveness of systemic propranolol supports the view that NE effects on gene expression in NCM are mostly through ADRAs.

Our data indicate that NE exerts a positive effect on song-induced gene expression, suggesting that NE may act through ADRA1s. These receptors are coupled to G_αq_-protein, which activates phospholipase C (PKC), leading to inositol triphosphate (IP3) and diacylglycerol release. Activation of this pathway could then increase intracellular Ca^+2 ^or further PKC activation, eventually resulting in activity-dependent gene induction. Alternatively, ADRA1 activation may activate Src family tyrosine kinases, which in turn activate ERK1/2 [42]. We found that ADRA1d is the most abundant ADRA in NCM and the main subtype expressed in song-responsive cells, while expression of ADRA2s, which are coupled to the inhibitory G_αi_-protein [43] and known to mediate NE suppressive effects in the song system [44], [45], is very low. We conclude that locally released NE most likely acts on ADRA1d to modulate the induction of gene expression in song-responsive NCM neurons.

### Noradrenergic Coupling of Electrophysiological and Gene Expression Responses

**Figure 7:**
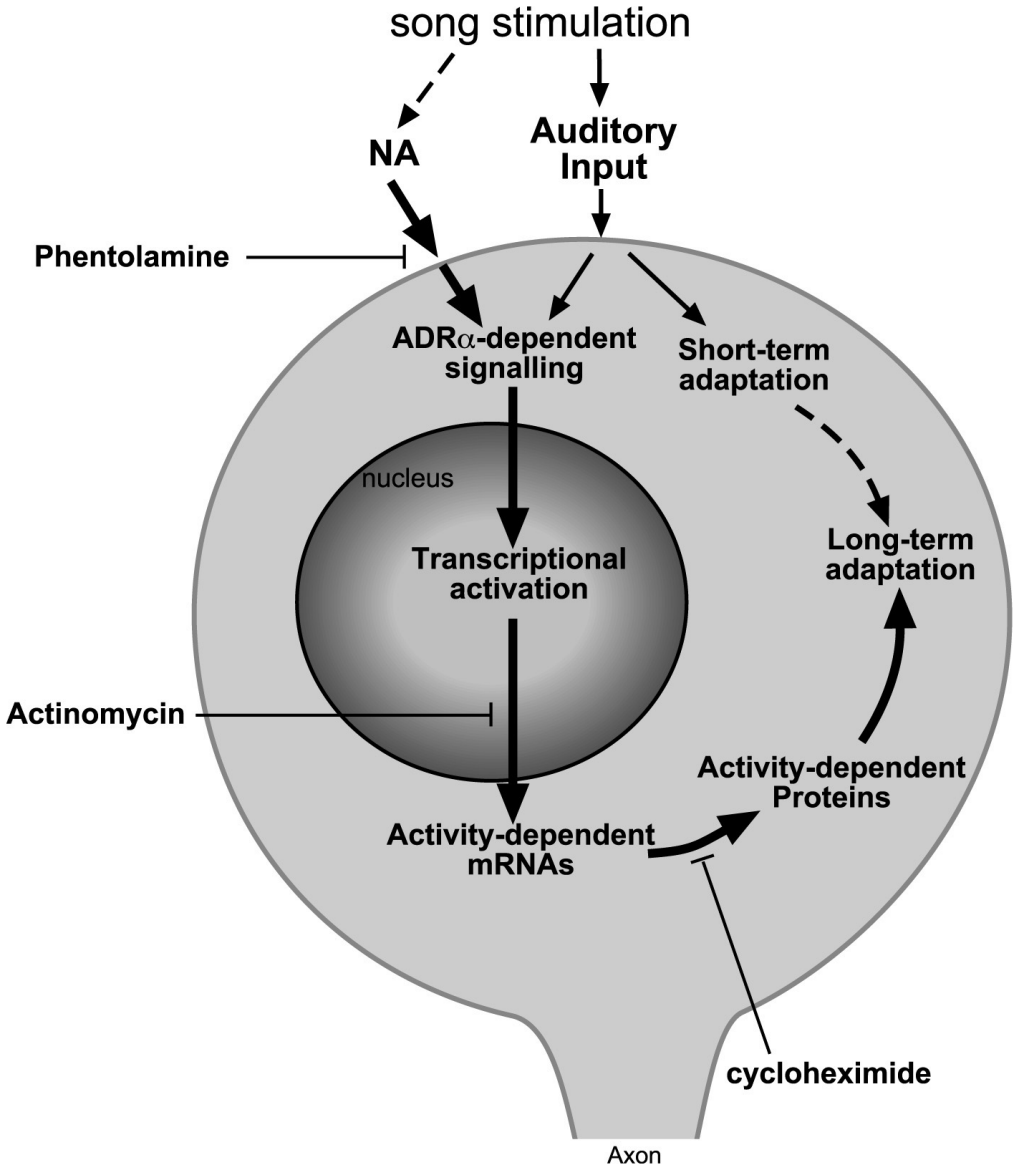
Noradrenergic modulation of song-induced gene expression and long-lasting changes in NCM auditory neurons. Undisturbed α-adrenergic transmission is required for song-induced gene expression and the long-term maintenance of song-adaptation yet does affect short-term habituation, indicating separate mechanisms. Blocking the downstream transcription (actinomycin) and translation events (cycloheximide) also results in disruption of long-lasting adaptation.

Locally-applied phentolamine interferes with gene induction without affecting NCM’s immediate electrophysiological responsiveness to song. This is a first demonstration that these two responses can be dissociated pharmacologically. Contrary to what occurs in some mammalian thalamic and cortical circuits [41] and in song nuclei of zebra finches [44], [45], [46], noradrenergic transmission at α-receptors does not seem to directly modulate the firing properties of NCM neurons. Rather, local α-adrenergic antagonism is able to dissociate the electrophysiological and genomic responses that normally occur in song-responsive neurons. The corollary is that these two responses are normally coupled, possibly through the activation of ADRAs concomitant to the auditory activation of NCM neurons. We propose a simple model (Fig. 7) whereby this could be accomplished. The model posits that inputs from the ascending auditory pathway and from noradrenergic projections, both activated by song auditory stimulation, converge onto song-responsive neurons and are required for song-induced gene expression. Supporting this model, NCM receives ample projections from preceding auditory centers [47] and contains a rich noradrenergic innervation in proximity to *zenk*-expressing cells, which co-express ADRAs (mainly *ADRA1d*). Since song-induced gene expression reflects the acoustic properties of the stimulus, the auditory input is an important determinant of induced gene expression and must interact with the noradrenergic input, but the nature of this interaction is unclear. Previous studies show that song-induced gene expression in NCM requires MAP kinase activation [16], [48], which could be downstream of ADRs through Ca^+2^−dependent cascades or Src. Our model may also explain how modulatory variables affect song-induced gene expression [e.g., a foot-shock enhances expression of song-induced genes but fails to induce them in NCM in the absence of auditory stimulation [34]. Furthermore, NE release from activated projections from the LoC would likely be maximal for stimuli of high attentional salience, explaining the higher levels of induced gene expression for novel than familiar songs.

### NE and Consolidation of Song-induced Cellular Changes

NCM responses to novel songs adapt in a manner that is long-lasting, song-specific, and dependent on song-induced gene expression for its long-term maintenance [20]. This experience-dependent change in song-responsive circuitry is a candidate cellular substrate for song auditory memories, but specific synaptic mechanisms are unclear. Our present data show that long-term adaptation depends on α-adrenergic transmission in NCM during the stimulation that induces adaptation. Since phentolamine did not affect spontaneous or song-evoked electrophysiological activity, this dependence is not due to noradrenergic modulation of NCM’s immediate electrophysiological response to song. Since phentolamine did not affect NCM’s short-term adaptation, it also appears that α-adrenergic transmission in NCM is not required for the acquisition of the adapted state. Considering the marked effects of phentolamine on gene expression, we conclude that α-adrenergic blockade during song stimulation prevents the gene induction events required for long-term adaptation. In other words, under normal conditions, the convergence of auditory and noradrenergic inputs triggers gene regulatory events that result in the consolidation of the adapted state in NCM (Fig 7).

Our conclusion resonates with findings in mammals. Adrenergic transmission is required for the maintenance of long-term potentiation (LTP) in the hippocampus after tetanic stimulation of the perforant pathway projection onto the dentate gyrus [49] or of mossy fibers onto CA3 cells [50], and in the lateral amygdala [51]. Direct pharmacological activation of the LoC leads to hippocampal synaptic plasticity as measured 24 hr later [10], and the maintenance of NE-dependent synaptic changes in the hippocampus and amygdala requires *de novo* RNA and protein synthesis [10], [50], [51]. Thus, distinct mechanisms underlie short and long-term synaptic plasticity, with NE playing a more significant role in the retention than the acquisition of this plasticity, an action likely mediated through regulation of gene expression.

Contrary to the tetanic stimulation used to induce LTP, birdsong is a natural learned stimulus and our gene induction paradigm uses awake freely-behaving animals. Thus, our results link noradrenergic transmission more directly to the neuronal plasticity underlying the response to a natural stimulus of behavioral relevance. Also contrary to LTP, adaptation reflects a decrease in neuronal responsiveness, perhaps more in line with long-term depression. Accordingly, NE acting through ADRA1 induces depression of hippocampal synapses that lasts ∼40 min, a form of LTD requiring both Src and ERK activity [52]. Thus, the noradrenergic system appears to play a facilitating role in long-term neuronal plasticity but not to determine the direction of the plastic change.

### NE and Memory Consolidation

The electrophysiological and gene expression responses in NCM are influenced by exposure of juveniles to tutor song, which serves as a reference for vocal learning, suggesting that a memory trace of tutor song is formed in the NCM of juveniles [31], [53]. Moreover, NCM lesions affect tutor song recognition [18] and local blockade of MEK1/2 activity, which is required for song-induced gene expression in NCM, disrupts song learning in juveniles [54]. These results implicate NCM in perceptual discrimination and memorization of tutor song. Given our present results linking NE to the expression of song-inducible genes and long-term adaptation, and the known involvement of NE in learning and memory, the noradrenergic system may also be an important regulator of cellular changes associated with tutor song memorization, a hypothesis that requires testing. Such an action would be distinct from the repressive alpha-2-mediated effects seen in motor nuclei of the song control [45].

We believe our data are also relevant to understanding the long-term effects of NE on learning and memory in general [4], [6], [7], [33]. We suggest that noradrenergic input induces gene regulatory programs in neurons activated during the acquisition of a given task, which then helps to consolidate the neuronal changes underlying the task memorization (akin to the cellular diagram on Fig 7). Interestingly, in contrast to NCM, the evidence in mammals points mostly to an involvement of β-adrenergic receptors in learning and memory [55]. While this discrepancy could reflect species differences, it could also relate to regional specializations: while the mammalian data derive mostly from the hippocampus and amygdala, NCM is a high-order avian auditory area analogous to supragranular layers of the mammalian auditory cortex [17]. A considerable body of evidence indicates that the learning-related NE effects in the pre-frontal cortex are predominantly through ADRAs [6]. Thus, α-adrenergic transmission may play a more prominent role in cortical-like circuitry than in the amygdala and/or hippocampus of vertebrates.

In sum, our results establish a prominent role for noradrenergic transmission in regulating both the molecular and electrophysiological responses to birdsong, a learned vocal behavior. More generally, they point to molecular and cellular mechanisms that may help explain the contribution of noradrenergic modulation to the memorization of sensory events, and suggest new directions that can be tested through further experimentation in both birds and mammals.

## Supporting Information

Figure S1 Noradrenaline induces activity-dependent gene expression in the NCM. a) Camera lucida drawing of a parasagittal brain section containing NCM at the level analyzed; b) Autoradiographic images of brain sections from birds that received local injections of saline or varying concentrations of noradrenaline hybridized with *zenk* riboprobes.
(TIF)

Table S1 Summary of the zebra finch alpha-adrenergic receptor genes and the conservation levels of the cloned fragments compared to chicken and human homologues.
(DOCX)
