# Supplementary figures and images for: Noradrenergic Control of Gene Expression and Long-Term Neuronal Adaptation Evoked by Learned Vocalizations in Songbirds
Source: PLoS One. 2012 May 4;7(5):e36276. doi: 10.1371/journal.pone.0036276 (PMC3344865; doi:10.1371/journal.pone.0036276)

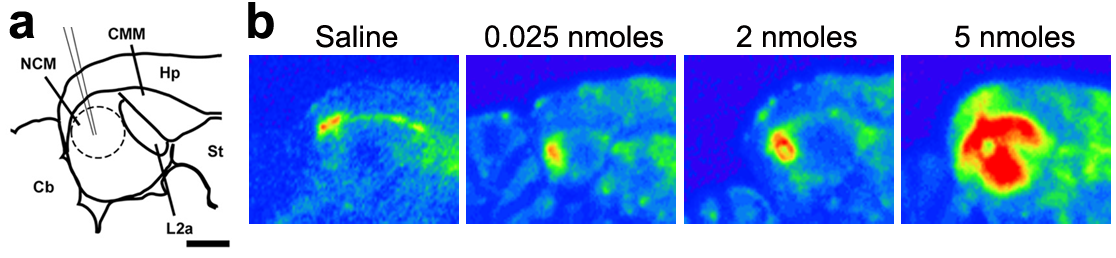

Supplement: Figure S1 — Noradrenaline induces activity-dependent gene expression in the NCM. a) Camera lucida drawing of a parasagittal brain section containing NCM at the level analyzed; b) Autoradiographic images of brain sections from birds that received local injections of saline or varying concentrations of noradrenaline hybridized with zenk riboprobes. (TIF) [file pone.0036276.s001.tif]
