# Supplementary material for: Noradrenergic Control of Gene Expression and Long-Term Neuronal Adaptation Evoked by Learned Vocalizations in Songbirds
Source: PLoS One. 2012 May 4;7(5):e36276. doi: 10.1371/journal.pone.0036276 (PMC3344865; doi:10.1371/journal.pone.0036276)
Supplement: Table S1 — Summary of the zebra finch alpha-adrenergic receptor genes and the conservation levels of the cloned fragments compared to chicken and human homologues. (DOCX) [file pone.0036276.s002.docx]

| **Gene Name** | **Ensembl Gene ID** | **Chr. #** | **Chr. location** | **Probe size**  **(bp; aa position*)** | **GenBank ID** | **Conservation**  **(% identity)** | |
| --- | --- | --- | --- | --- | --- | --- | --- |
|  |  |  |  |  |  | **Chicken** | **Human** |
| Alpha-adrenergic receptor 1a | ENSTGUT00000004327 | 22 | 1321711-1331853 | 687 (aa 107-339) | pending | 92.1 | 60.7 |
| Alpha-adrenergic receptor 1b | ENSTGUT00000000212 | 13 | 1255831-1268052 | 523 (aa 121-294) | pending | 84.9 | 87 |
| Alpha-adrenergic receptor 1d | ENSTGUT00000011389 | 4 | 68626540-68670188 | 435 (aa 97-240) | pending | 97.9 | 78.6 |
| Alpha-adrenergic receptor 2a | ENSTGUT00000011150 | 6 | 26060001-26061188 | 704 (aa 130-395) | pending | 92.7 | 67 |
| Alpha-adrenergic receptor 2b | [ENSTGUT00000000407](http://www.ensembl.org/Taeniopygia_guttata/transview?transcript=ENSTGUT00000000407) | 13 | 3,406,160-3,407,032 | 296 (aa 6-85) | pending | 89.7 | 71 |
| Alpha-adrenergic receptor 2c | ENSTGUT00000010756 | 4 | 61351183-61352421 | 1031 (aa 93-427) | pending | 97 | 77.8 |

**Table 1.** Summary of the zebra finch alpha-adrenergic receptor genes and the conservation levels of the cloned fragments compared to chicken and human homologues.

*aa position refers to the amminoacid position in relation to the human homologue; Chr., chromosome.
